# Active Breaks in Primary and Secondary School Children and Adolescents: The Point of View of Teachers

**DOI:** 10.3390/healthcare13192482

**Published:** 2025-09-29

**Authors:** Michela Persiani, Andrea Ceciliani, Gabriele Russo, Laura Dallolio, Giulio Senesi, Laura Bragonzoni, Marco Montalti, Rossella Sacchetti, Alice Masini

**Affiliations:** 1 Department for Life Quality Studies, University of Bologna, 47921 Rimini, Italy; michela.persiani3@unibo.it (M.P.); andrea.ceciliani@unibo.it (A.C.); giulio.senesi2@unibo.it (G.S.); laura.bragonzoni4@unibo.it (L.B.); 2 Department of Economics, Engineering, Society and Business Organization, University of Tuscia, 01100 Viterbo, Italy; 3 Department of Biochemical and Neuromotor Sciences, University of Bologna, 40126 Bologna, Italy; laura.dallolio@unibo.it; 4 Unit of Hygiene and Public Health Forlì-Cesena, Department of Public Health, Romagna Local Health Authority, 47522 Cesena, Italy; marco.montalti9@unibo.it; 5 Department of Medical and Surgical Sciences, University of Bologna, 40138 Bologna, Italy; 6 Department of Education Studies, University of Bologna, 40126 Bologna, Italy; rossella.sacchetti@unibo.it; 7 Department of Translational Medicine, University of Eastern Piedmont, 28100 Novara, Italy; alice.masini@uniupo.it

**Keywords:** active breaks, teacher perceptions, classroom physical activity, sedentary behavior, learning environment, school environment

## Abstract

**Background/Objectives:** Engaging in regular physical activity (PA) and reducing sedentary behaviors benefits youth health, especially for those with disabilities. However, two-thirds of European children remain insufficiently active. In schools, Active Breaks, brief 5–15 min PA sessions led by teachers during or between lessons, offer a feasible strategy to increase movement. This study investigated teachers’ perceptions of ABs by comparing implementers and non-implementers, examining facilitators and barriers to implementation, and exploring their potential to support the inclusion of students with disabilities. **Methods:** An observational cross-sectional study was conducted among primary and secondary school teachers in the Emilia-Romagna region (Italy), all of whom had completed a 6 h training course on the implementation of ABs. Data were collected using an ad hoc questionnaire consisting of four sections: sociodemographic data, an adapted Attitudes Toward Physical Activity scale, ABs’ practicality/sustainability, and inclusiveness. **Results:** Overall, 65% of teachers reported implementing ABs, with higher adoption in primary (69.5%) than secondary schools (58.6%). Implementers reported more positive perceptions and attitudes across individual, classroom, and school-support domains (*p* < 0.05). In addition, primary teachers consistently scored higher than their secondary counterparts, particularly in terms of class characteristics and benefit perceptions (*p* < 0.001). Most teachers, especially in primary schools (84.2%), reported no difficulties, although one-third of secondary teachers reported challenges. Exploratory factor analysis on barrier items identified two dimensions (practical/logistical feasibility; institutional/procedural difficulties), but internal consistency was low. Teachers mainly reported using motor activities, with sessions lasting 5–10 min, typically scheduled mid-morning. Inclusion analysis revealed that 60% of teachers had students with disabilities in their classes. While most students participated without adjustments, 25% required occasional or consistent modifications. **Conclusions:** ABs are a practical and inclusive strategy to reduce SBs in schools. However, not all teachers are currently able to implement them, due to varying contextual constraints, levels of support, and perceived barriers. Primary school settings appear more conducive to their integration, whereas secondary schools may require more tailored support. Fostering teacher confidence, peer collaboration, and inclusive planning can enhance both the implementation and long-term sustainability of educational initiatives.

## 1. Introduction

Engaging in regular physical activity (PA) and reducing sedentary behaviors (SBs) are widely recognized as beneficial for the physical and mental health of children and adolescents [1,2], especially for those with disabilities [3,4]. Specifically, PA offers advantages across multiple health domains, including improved physical fitness (cardiorespiratory and muscular fitness), enhanced cardiometabolic health (blood pressure, dyslipidemia, glucose regulation, and insulin sensitivity), stronger bone health, better cognitive outcomes (academic performance and executive function), improved mental health (reduced symptoms of depression), and decreased adiposity [1,5]. Conversely, higher levels of SB in this population are associated with adverse health outcomes, such as increased adiposity, poorer cardiometabolic health and fitness, reduced sleep duration, and negative impacts on behavioral regulation and prosocial functioning [5].

Alarmingly, two-thirds of European children and adolescents are not sufficiently active, with a clear trend of lower activity levels in Southern compared to Northern regions [6]. This highlights the need for policymakers, governments, and stakeholders at both local and national levels to implement structural and political changes to promote PA and reduce SB among European youth [6]. Although SBs and physical inactivity are sometimes used interchangeably, they are distinct constructs that are necessarily correlated, and both carry significant health risks [7,8]. Physical inactivity, defined as not achieving the recommended average of 60 min per day of moderate-to-vigorous intensity physical activity for children and adolescents aged 5–17 years, has often been referred to as a 21st-century epidemic [9,10,11]. By contrast, SBs refer to any waking behavior characterized by an energy expenditure ≤ 1.5 metabolic equivalents (METs), while in a sitting, reclining, or lying posture, such as TV viewing, video game playing, computer use (collectively termed “screen time”), or sitting in a car [12]. Identifying opportunities for children and adolescents to achieve the recommended 60 min of moderate-to-vigorous PA daily is crucial for enhancing fitness, developing movement skills, and preventing chronic diseases [13]. These challenges are even more pronounced among children with disabilities (e.g., intellectual and/or physical), who often fail to achieve sufficient PA, accumulate most of their activity at school, and show limited moderate-to-vigorous physical activity (MVPA) during physical education and recess [14,15]. High levels of overweight and obesity, poorer motor skills, and reduced participation in structured activities further hinder their engagement in PA [3,16,17]. Similarly, children with autism spectrum disorder (ASD) and/or attention-deficit/hyperactivity disorder (ADHD) face additional barriers such as motor coordination, sensory, and attentional difficulties, which contribute to higher SB and lower PA levels [18,19,20].

Schools play a key role in promoting health by interrupting SBs and integrating PA into daily routines through before- and after-school programs, physical education classes, and active breaks during recess and lunch [21]. While integrating PA helps to reduce sedentary time, its implementation often competes with academic demands, limiting widespread adoption. Efficient and adaptable strategies are therefore needed to balance these competing priorities and to support the integration of physical activity within the school setting [22,23].

In this regard, School Active Breaks (ABs) have emerged as a practical and effective strategy for incorporating PA into educational settings. ABs are short sessions of PA, typically lasting 5 to 15 min, led by appropriately trained teachers, and conducted during daily educational settings [24]. These short bursts of movement help interrupt prolonged sitting while fitting seamlessly into the curricular schedule [25]. Evidence shows that ABs can increase PA levels, improve classroom behavior (e.g., on-task behavior [26,27,28,29]) and working memory, and encourage a positive attitude toward movement and exercise [30]. Moreover, a recent systematic review indicated that ABs help children and adolescents to rediscover the pleasure of moving and reach the minimum level of PA recommended for health [31,32].

Significantly, the effectiveness of ABs is reinforced by teachers’ positive perceptions [22,33,34]. This favorable outlook underscores the importance of teacher engagement and training in successfully implementing ABs within educational settings. However, previous studies have emphasized that, for ABs to be effective, they must be brief, quick to execute, adaptable to the limited space available in classrooms, and easy to implement without requiring sophisticated equipment [33,35]. When properly designed and implemented, ABs are strongly supported by teachers. ABs are often perceived as a valuable tool for enhancing students’ physical activity, focus, and conflict management in the class within the constraints of the school day [30,36]. Despite these benefits and their importance within the educational community, ABS are still underutilized in schools [30,36]. The potential of ABs may be particularly relevant for children with disabilities, who often face lower PA levels, higher sedentary behavior, and additional barriers to participation compared to their typically developing peers. For example, children with intellectual disabilities (ID) often exhibit deficits in working memory, especially in the phonological loop [37], while children with ASD or ADHD may experience motor coordination, sensory, or attentional difficulties that limit their engagement in PA. In these populations, ABs could serve not only to interrupt sedentary time but also as a supportive educational practice with potential cognitive and social-behavioral benefits [3].

In Italy, the National Prevention Plan 2020–2025 promotes health-enhancing school environments, particularly via the Program “Schools that Promote Health” [38]. Within this framework, the Emilia-Romagna region has identified a list of recommended practices for schools promoting health, including ABs, as practical tools to counteract sedentary behavior in children and adolescents. While previous literature supports the positive effects of ABs, questions remain regarding the facilitators and barriers that influence teachers’ decisions to implement these breaks. Further research is needed to understand the factors that shape teachers’ ability and willingness to adopt ABs. Identifying these factors could foster greater acceptance among teachers and stakeholders, leading to more widespread adoption.

The present study aims to explore how teachers across different school levels perceive the role of ABs in terms of their benefits, facilitators, and barriers, by comparing implementers and non-implementers. It also investigates the potential of ABs to support the inclusion of pupils with disabilities. The findings are intended to inform strategies for effective and sustainable implementation. We hypothesized that teachers would generally view ABs as beneficial and feasible within the school environment. However, secondary school teachers might face greater challenges in implementation compared to primary school teachers. Additionally, we anticipated the identification of specific barriers that could affect the applicability and sustainability of ABs, and we expected ABs to emerge as a supportive practice for fostering more inclusive educational settings for students with disabilities.

## 2. Materials and Methods

### 2.1. Sampling

A total of 481 teachers (447 females) were recruited across different school grades. Of the full sample, 300 teachers (293 females) worked in primary schools, and 181 (154 females) in secondary schools (middle and high school combined). Participants taught a variety of subjects, including mathematics, sciences, geography, chemistry, and informatics.

Teachers expressed their willingness to participate by contacting their school managers and subsequently attending a 6 h training program on implementing ABs. The training was delivered in one session of six hours each and systematically integrated theoretical foundations with practical application. The first part introduced the scientific rationale and benefits of ABs, providing participants with principles and guidance for their implementation The second was dedicated to hands-on practice, with instructors modeling simple, adaptable strategies to foster teachers’ skill-building, confidence, and self-efficacy. Following the training phase, each school formally agreed to participate in the project. Teachers were asked to implement ABs at least three times per day in each class, with complete discretion over content, timing, and pedagogical style. These elements were intentionally kept flexible to allow adaptation to class context, student needs, and time constraints.

### 2.2. Study Design

The study was an observational cross-sectional study conducted in accordance with the Survey Reporting Guideline (SURGE, [39]). Local Emilia-Romagna health agencies organized the training sessions by coordinating with school managers. The survey was conducted 3 to 6 months after the training session, allowing teachers sufficient time to decide whether to implement and integrate ABs in their classrooms.

Data collection was carried out via a census between 1 May 2024, and 31 July 2024. Participation in the survey was voluntary and pseudo-anonymous. Respondents were informed about the study’s objectives and provided informed consent prior to participation. No directly identifying information was collected, and unique codes were used to protect individual identities while allowing linkage of responses. This approach enhanced data reliability for sensitive topics while ensuring participant confidentiality [40]. Consent to participate was obtained in electronic format before survey administration. Participants had to read, understand, and accept all the information reported on the introduction page of the online survey.

The study was approved by the Ethics Committee of the University of Bologna (Ref. 0126773, 7 May 2024) and conducted in accordance with the Declaration of Helsinki.

### 2.3. Survey Questionnaire and Administration

The questionnaire named “The schools promoting health through ABs: teachers’ perceptions” was developed ad hoc with items organized into five sections:

1. Teacher sociodemographic characteristics (4 items collecting data such as gender, teaching role, years of experience, and school level).
2. Adapted version of the Attitudes Towards Physical Activity (ATPA) questionnaire evaluating teachers’ perceptions and attitudes toward ABs (11 items) and additional items assessing potential barriers (8 items) [41,42].
3. Practicability, applicability, sustainability of ABs (9 items) and perceived benefits experienced during their use (7 items).
4. Inclusion and disability (3 items, including those with special educational needs or diverse abilities).

Section 1 and Section 2 were completed by all teachers, including both implementers and non-implementers of ABs. Section 3 and Section 4 were specifically designed for teachers who had implemented ABs in the classroom. The items were selected through a critical review of the literature to define the specific items of each section [21,41,43]. After the initial development, cognitive interviews were conducted with a subsample of teachers to verify the clarity and interpretation of the item [44]. Based on their feedback, some items were revised to improve comprehension. The survey included multiple-choice questions rated on a 5-point Likert scale (1 = strongly disagree, 2 = disagree, 3 = neutral, 4 = agree, 5 = strongly agree).

The survey was promoted via email through Local health agencies and School representatives of the Emilia-Romagna region (Italy) and was administered using a dedicated online survey platform. This survey was accessible from both mobile devices and computers. A designated researcher monitored data collection and maintained access to the anonymized dataset. The study respected the anonymity and privacy of data in accordance with the General Data Protection Regulation (GDPR Regulation EU 2016/679) [45]. Answers were anonymized, and IP addresses were not visible to the researcher.

### 2.4. Statistical Analysis

Data were analyzed using R (v. 4.5.1) through the RStudio platform (v. 2025.09.0+38). Descriptive statistics were computed to summarize the variables. Dichotomous and categorical data were presented as frequencies and percentages, whereas continuous variables were expressed as means and standard deviations.

For the questionnaire data, items were grouped according to the three main sections of the survey. The internal consistency of each section was assessed using Cronbach’s α, which evaluates how consistently the items within a section measure the same construct.

Items related to barriers were examined through an exploratory factor analysis (EFA) to identify potential latent variables. Composite scores for each section were then computed by summing the respective items.

Linear mixed-effects models, suitable for non-parametric data (using the ARToolpackage, v. 0.11.2), were used to analyze associations. Data normality was assessed using the Shapiro–Wilk test. Independent variables of the models were “school grade” (primary vs. secondary) and the “implementation of ABs” (ABs; Implementers vs. Non-Implementers). When appropriate, post hoc analyses with Tukey corrections were conducted using the emmeans package (v. 1.11.2-8). Results are reported as F values with associated degrees of freedom, F(df1, df2), where df1 represents the numerator degrees of freedom and df2 the denominator degrees of freedom.

## 3. Results

### 3.1. Sociodemographic Characteristics

**Figure 1:**
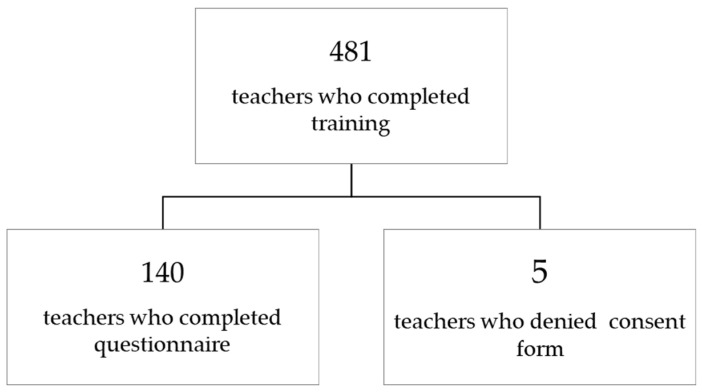
Participant flow diagram.

Of the 481 teachers who completed the training, 140 completed the questionnaire, and five refused to sign the consent form (Figure 1). Therefore, the present analysis is based on a subsample of 140 teachers. The characteristics of teachers are shown in Table 1.

Although the response rate in the present study was approximately 30%, this value is within the acceptable range reported in educational and organizational research, where typical rates range from 34% to 53% [46].

### 3.2. Teacher ABs Implementation

**Figure A1:**
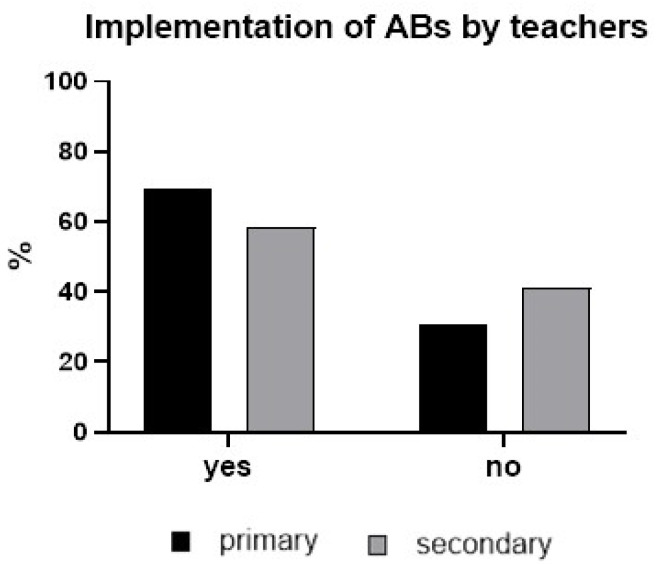
Implementation of ABs by teachers. Percentage of primary and secondary school teachers who implemented active breaks (ABs) during the school year.

Ninety-one teachers (65%) reported implementing ABs during the school year. Specifically, 57 (69.5%) primary school teachers stated they regularly integrated ABs into their teaching practice, compared to 34 (58.6%) secondary school teachers. Despite these encouraging numbers, a non-negligible portion of teachers reported not implementing ABs (see Figure A1 in Appendix A).

#### 3.2.1. Teachers’ Perceptions and Attitudes Toward ABs in the Classroom

Cronbach’s α for the Individual Perception variable was 0.91. The linear regression indicated that the AB factor was significant (F(1,136) = 41.78, *p* < 0.001, ƞ^2^ = 0.24). A higher score for Implementers compared to Non-Implementers was found (see Table 2). The “school grade” factor was also significant (F(1,136) = 8.83, *p* = 0.004, ƞ^2^ = 0.06) with primary school teachers reporting higher scores than secondary school teachers (M = 16.15, SD = 3.40 vs. M = 15.22, SD = 2.58 points). Interaction “ABs” × “school grade” was non-significant (F(1,136) = 0.17, *p* > 0.05, ƞ^2^ = 0.00).

**Figure 2:**
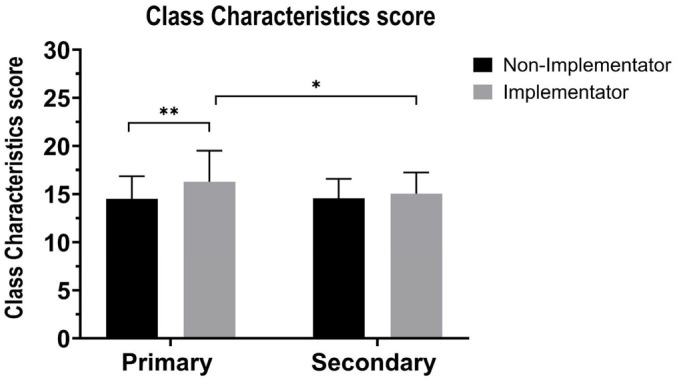
Class characteristics scores in primary and secondary classes for Implementers (gray bars) and Non-Implementers (black bars). Y-axis: class characteristics score; X-axis: school level. Class characteristics scores were calculated as the sum of responses within the domain. Mean (±SD) values are shown for Primary vs. Secondary students and for Implementors vs. Non-Implementors. Significant differences are indicated as follows: * *p* < 0.05; ** *p* < 0.01.

Cronbach’s α for the Class Characteristics variable was 0.79. Linear regression indicated the “ABs” factor was significant (F(1,136) = 35.35, *p* < 0.001, ƞ^2^ = 0.21), with a higher score for Implementers compared to Non-implementers (Table 2). “School grade” factor was also significant (F(1,136) = 18.26, *p* > 0.001, ƞ^2^ = 0.12). The score was higher for primary school teachers than for secondary school teachers (M = 11.72, SD = 2.22 vs. M = 10.52, SD = 1.95 points). The interaction “ABs” × “school grade” was nonsignificant (F(1,136) = 0.27, *p* > 0.05, ƞ^2^ = 0.00) see Figure 2.

Cronbach’s α for the Supporting the School Education variable was 0.88. Linear regression highlighted that the “ABs” factor was significant (F(1,136) = 9.16, *p* = 0.003, ƞ^2^ = 0.06). Analysis showed that Implementers had a higher score compared to Non-implementers (Table 2). The “School grade” factor was also significant (F(1,136) = 5.39, *p* > 0.001, ƞ^2^ = 0.04). Primary school teachers had a higher score than secondary school teachers (M = 15.75, SD = 3.07 vs. M = 14.86, SD = 2.13 points). The interaction “ABs” × “school grade” was significant (F(1,136) = 3.93, *p* > 0.04, ƞ^2^ = 0.03). Post hoc analyses revealed a significant difference between the primary school teachers who did not implement ABs and the primary school teachers who implemented ABs (t(136) = 3.55, *p* = 0.003, *d* = 0.61). No significant difference was observed between the primary school teachers who did not implement ABs and the secondary school teachers who did not implement ABs (t(136) = 0.07, *p* > 0.05, *d* = 0.01) or between the Non-Implementer primary school teachers and the Implementer secondary school teachers (t(136) = 0.72, *p* > 0.05, *d* = 0.12). No significant difference was found between Implementers and Non-Implementers of secondary school (t(136) = 0.79, *p* > 0.05, *d* = 0.14).

#### 3.2.2. Perceived Difficulties and Barriers to ABs

**Figure 3:**
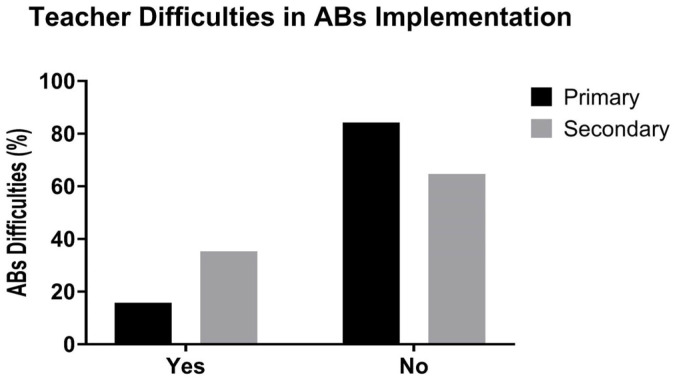
Teacher difficulties in ABs implementation in Primary (black bars) and Secondary (gray bars) schools. The y-axis represents the percentage of teachers reporting difficulties with ABs implementation, while the x-axis indicates whether teachers reported experiencing difficulties (Yes vs. No). Data are reported as percentages.

The analysis of perceived difficulties in implementing ABs revealed differences between primary and secondary school teachers. Most primary teachers reported no difficulties (84.2%), compared with 64.7% of secondary teachers, among whom 35.3% experienced challenges (Figure 3).

Items related to barriers associated with the implementation of ABs; an EFA was conducted on the six items assessing barriers to the implementation of ABs (Table 3). The internal consistency of the items was low (Cronbach’s α = 0.35), and the Kaiser–Meyer–Olkin (KMO) measure of sampling adequacy indicated a poor overall fit (KMO = 0.56), although all individual Measure of Sampling Adequacy values were above the 0.50 threshold. Bartlett’s test of sphericity was significant (χ^2^(15) = 97.11, *p* < 0.001), indicating that the correlation matrix was suitable for factor analysis. Parallel analysis suggested the extraction of two factors. The two-factor model with varimax rotation showed good model fit (χ^2^(4) = 1.21, *p* = 0.877) and accounted for 45% of the total variance (Factor 1 = 28%; Factor 2 = 17%). Factor 1 was strongly defined by BARR3 (0.82) and BARR7 (1.00), whereas Factor 2 was characterized by BARR6 (0.67), BARR2 (0.48), and BARR5 (–0.47), with BARR1 showing a weaker loading (0.31). The uniqueness values indicated that BARR1 (0.90) and BARR5 (0.78) contributed less to the overall factor structure. Overall, these results suggest a two-factor solution, reflecting practical/logistical feasibility and institutional/procedural challenges as the main latent dimensions of perceived barriers. However, the relatively low internal consistency highlights the need to refine and validate the barrier items in future research, possibly by clarifying wording or developing additional items to strengthen each dimension.

Cronbach’s α for Factor 1 was 0.90. The linear regressions did not highlight any significant differences for all the factors and the possible interaction (F < 2.95, *p* > 0.05). Cronbach’s α for Factor 2 was 0.04, and the linear regressions did not highlight any significant differences for all the factors and the possible interaction (F < 0.55, *p* > 0.05).

### 3.3. Practicability, Benefits, Applicability, and Sustainability of ABs in the Classroom

The profile of teachers who implemented ABs varied between primary and secondary school levels (see Table A1 in Appendix A). Among primary school teachers, 44 (77.19%) reported “Definitely Yes” about whether they were comfortable suggesting ABs, compared to 24 (70.59%) secondary school teachers. In contrast, only 1 (1.75%) primary school teacher indicated discomfort (i.e., “More NO than YES”), compared to 2 (5.88%) at the secondary level. Winter (December–February) was the most common starting period for ABs in both groups, reported by 23 teachers in each group (40.35% of primary and 67.65% of secondary teachers). Among primary school teachers, 29 (50.88%) performed ABs a few times a week, while 11 (19.30%) reported doing so several times a day and 12 (21.05%) once daily. In contrast, among secondary school teachers, 22 (64.71%) reported performing ABs a few times a week, and only 5 (14.71%) managed to conduct them once a day. Regarding duration, 34 (59.65%) of primary school teachers and 14 (41.18%) of secondary school teachers reported that ABs typically lasted 10 min, followed by 5 min sessions, reported by 20 (35.09%) and 15 (44.12%) teachers, respectively. Teachers identified mid-morning as the most helpful time for ABs, with 25 (43.86%) primary and 26 (76.47%) secondary teachers selecting this period. In terms of content, “Motor” activities were the most frequently used in both groups: 39 (68.42%) in primary and 23 (67.65%) in secondary. Mixed content was used by 14 (24.56%) primary and 6 (17.65%) secondary teachers, while cognitive content was used exclusively in secondary schools by two teachers (5.88%). Leadership of ABs was most commonly assumed by the teacher, with 38 (66.67%) primary and 14 (41.18%) secondary school teachers leading the breaks. However, secondary schools involved pupils more frequently, with 8 (23.53%) ABs led entirely by students and 9 (26.47%) co-led by teachers and students. The use of digital tools, particularly interactive whiteboards, was limited. Among primary school teachers, 8 (14.04%) reported using the interactive whiteboard during ABs, compared to 6 (17.65%) of secondary teachers. YouTube was the most used application, cited by four teachers in primary (7.02%) and six in secondary (17.65%), followed by “mixed tools” (7.02% and 5.88%, respectively). These findings highlight some differences between school levels in terms of frequency, modality, leadership, and technological support for ABs, with greater challenges and variability reported in secondary school settings.

**Figure 4:**
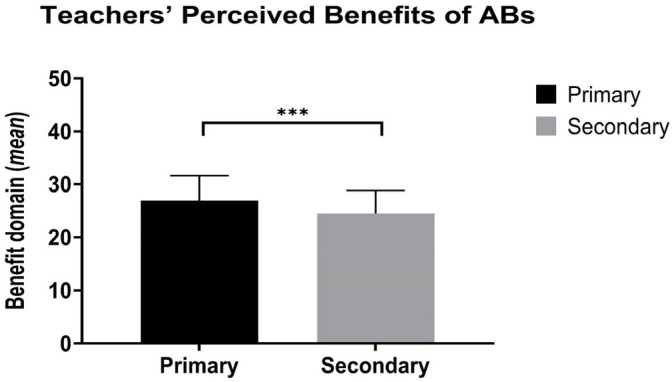
Teachers’ perceived benefits of ABs in Primary (black bars) and Secondary (gray bars) schools. The y-axis represents the mean score of perceived benefits, while the x-axis indicates school level (Primary vs. Secondary). Data are reported as mean values of the benefit domain score (±standard deviation). Significant differences are marked as follows: *** *p* < 0.001.

Cronbach’s α for the teachers’ perceptions of the benefit of ABs was 0.92. Linear regression showed that “school grade” factor was significant (F(1,89) = 13.73, *p* < 0.001, ƞ^2^ = 0.13), highlighting a higher score for primary school teachers compared to secondary school teachers (Figure 4 and Table 4).

### 3.4. Inclusion and Disability

The survey revealed that the majority of teachers had students with disabilities in their classrooms, with 34 (59.65%) primary school teachers and 22 (64.71%) secondary school teachers confirming their presence (see Table A2 in Appendix A). A small proportion of respondents preferred not to disclose this information (5.26% in primary and 5.88% in secondary schools). Among those who specified the type of disability, cognitive disabilities were the most frequently reported, noted by 27 (47.37%) primary and 15 (44.12%) secondary school teachers. Physical disabilities were reported by 2 (5.88%) teachers in secondary schools only, while sensory disabilities were mentioned by 2 (3.51%) primary school teachers. Combined disabilities, such as cognitive and physical, were reported by one teacher in each school level (1.75% in primary and 2.94% in secondary), whereas cognitive and sensory disabilities were reported by 2 (3.51%) teachers in primary school only. A small group of respondents (3.51% primary and 11.76% secondary) preferred not to specify the type of disability. Regarding the need to adjust the timing, rhythms, or scheduling of ABs to meet the needs of students with disabilities, responses were varied. In primary schools, 11 teachers (32.25%) reported that adjustments were never needed, while 10 (29.41%) indicated that they were sometimes required. Smaller proportions reported adjusting almost never (23.53%), always (11.76%), or rarely (2.94%). In secondary schools, 10 teachers (45.45%) stated that no adjustments were needed, 6 (27.27%) reported adjusting sometimes, and only 2 (9.09%) indicated constantly adjusting ABs. The responses “almost never” and “rarely” were each selected by two secondary teachers (9.09%). These findings highlight the relevance of inclusive planning when designing and implementing active breaks. Although the need for adjustments was not universal, a non-negligible proportion of teachers reported modifying ABs to support students with disabilities, underscoring the importance of flexibility and responsiveness in school-based physical activity interventions.

## 4. Discussion

This study examined the relationship between teachers’ self-reported implementation of ABs, their perceptions and attitudes, perceived barriers and benefits, and school levels, in order to identify strategies that support effective and sustainable adoption.

The sociodemographic profile of the sample offers insights into the profile of teachers engaged in ABs. The predominance of female respondents, typical in the Italian school system due to the high proportion of female teachers, reflects demographic trends observed in previous European studies, where teaching staff is often predominantly female and highly experienced [47,48]. These characteristics may influence attitudes and practices related to the promotion of PA in the classroom.

Our analysis revealed important differences in AB implementation between primary and secondary school teachers. While the majority of teachers reported integrating ABs into their teaching routines, implementation rates were higher in primary schools. This pattern suggests that curriculum flexibility and pedagogical approaches may facilitate integration at this educational level. Primary school curricula often emphasize holistic child development, incorporating PA and playing embedded in daily learning experiences [48]. In contrast, secondary school teachers reported greater challenges, likely due to subject-specific instructional demands, time-stricter lesson schedules, and limited curricular flexibility [49,50].

Perceptions, attitudes, beliefs, and self-efficacy regarding ABs implementation, assessed through a modified version of the ATPA [41,42], showed that teachers who implemented ABs reported more positive perceptions and attitudes across individual, classroom, and school support domains. This finding suggests a possible increase in self-efficacy and the perceived feasibility of applying ABs in everyday teaching practice [25,50,51], corroborating previous evidence that teacher confidence and motivation are critical facilitators of classroom-based physical activity interventions [48,52]. Classroom-level factors, such as students’ behavior and perceived time availability, also influenced the implementation of ABs, in line with literature identifying classroom management and time constraints as recurrent determinants of PA adoption [53]. Notably, primary school teachers consistently scored higher across domains than secondary school teachers, reinforcing the idea that contextual and organizational differences strongly shape implementation feasibility.

The last social–ecological dimension of school support further emerged as a key facilitator. Teachers who perceived strong institutional and peer support were more likely to adopt ABs, supporting previous findings that highlight the importance of leadership and school culture in sustaining classroom-based practices [22,33].

Despite these generally favorable perceptions, some barriers were reported. Most primary school teachers described few or no difficulties, whereas more than one-third of secondary teachers perceived challenges, reflecting the contextual differences between the two educational levels. Exploratory factor analysis of the barrier items indicated two main dimensions: practical/logistical feasibility (e.g., space, materials, class size, and time) and institutional/procedural challenges (e.g., bureaucracy, training, and organizational support). Although the internal consistency of the barrier scale was low, these categories align with established literature that identifies a lack of time, limited space, and classroom management as common barriers [35,53]. Interestingly, in our sample, perceptions of training adequacy did not differ between implementers and non-implementers, suggesting that the training provided may have equipped teachers with sufficient knowledge and confidence to deliver ABs effectively.

Overall, teachers perceived ABs as feasible and beneficial in classroom practice, although implementation patterns varied between primary and secondary schools. Primary school teachers tended to deliver ABs more frequently, whereas secondary school teachers provided shorter and less frequent sessions, likely constrained by organizational and time-related factors. Across both levels, motor-based activities were the most frequently adopted, indicating a preference for simple movement tasks over cognitively integrated ones. Interestingly, secondary schools more often involve students in leading ABs, a strategy that may enhance engagement and sustainability in older age groups [50]. Benefit scores were significantly higher among primary school teachers, reinforcing the notion that primary education contexts are more conducive to the integration of ABs.

The potential of ABs as an inclusive practice also emerged as a key finding. Students with disabilities typically engage in lower levels of PA during school hours [54]. ABs can help address this gap by offering accessible opportunities for movement. Previous research, such as that conducted by Mazzoli et al. [3], has shown that ABs can provide moderate cognitive benefits and reduce sedentary behavior among students with intellectual disabilities, while also highlighting potential challenges, such as overstimulation in students with ASD. These findings underscore the importance of teacher training and tailored support, including the use of visual aids and adapted instructional strategies.

Our survey further confirmed the potential of ABs for inclusion. A substantial proportion of teachers reported having students with disabilities in their classes (59.65% in primary and 64.71% in secondary schools), primarily with cognitive impairments. While some students participated without adjustments (19.30% in primary, 29.41% in secondary), others required occasional (17.5%) or consistent (7%) modifications. In many secondary schools, the presence of physical education teachers with a degree in Sports Science may have facilitated adaptation. These findings suggest the importance of a flexible, tailored approach to ensure ABs are accessible to all students. While cognitively demanding motor tasks can enhance engagement among typically developing students, they may pose challenges for students with neurodevelopmental disorders [55]. This emphasized the need for balanced task design, guided instructions, adapted equipment, and peer-assisted activities [55,56]. By ensuring that ABs accommodate diverse needs, schools can foster more inclusive environments, promote PA, and reduce disparities in movement opportunities and health outcomes [56]. This aligns with broader educational equity goals and supports the development of healthier, more inclusive school cultures. Research on the intersection of ABs and inclusivity remains limited, and our findings provide valuable new perspectives. While ABs can foster inclusion and cognitive engagement, their effectiveness depends heavily on thoughtful design and implementation. Future studies should incorporate qualitative and observational approaches to better understand the real-world adaptability of ABs and to develop evidence-based strategies that maximize accessibility and engagement for all students.

Several limitations of this study should be acknowledged. The relatively low response rate, influenced by formal recruitment and distribution procedures, may limit the generalizability of the findings. The self-reported nature of the data could also introduce response bias, as perceptions and reported practices may not fully reflect actual implementation. Additionally, although preliminary analyses supported the reliability of the questionnaire (e.g., internal consistency and exploratory factor analysis), the tool has not been fully validated and should be refined in future research. Finally, the cross-sectional design captures only a snapshot of teachers’ practices and perceptions, precluding conclusions about longitudinal trends or causal relationships. Future research should employ representative, longitudinal designs, validate the questionnaire through robust psychometric testing, and examine contextual factors that influence the adoption and sustainability of ABs.

## 5. Conclusions

ABs represent a promising and accessible strategy to foster PA, cognitive engagement, and inclusion within school settings. When thoughtfully designed and adequately supported, ABs can offer significant benefits for all students, particularly those with disabilities, by promoting participation, reducing sedentary behavior, and enhancing classroom learning environments. To unlock their full potential, comprehensive teacher training, strong institutional commitment, and inclusive, adaptable implementation strategies are essential across all educational levels.

## Figures and Tables

**Table 1:** Characteristics of teachers.

(N = 140)	N	%
**Gender**		
Female	123	87.9
Male	17	12.1
**Level of education**		
No graduate degree	24	17.1
Bachelor’s and master’s degrees	116	82.9
**Years of experience as a teacher**		
1–10	46	32.9
11–20	44	31.4
+21	50	35.7
**School type**		
Primary School	82	58.6
Middle and High school	58	41.4

Number of observations N. and percentage %.

**Table 2:** Teacher perceptions and attitudes.

	Non-Implementers (N = 49) M ± SD	Implementers (N = 91) M ± SD
INDIVIDUAL PERCEPTIONS *	14.16 ± 2.63	16.63 ± 3.01
Feels able to conduct ABs with students		
Feels motivated to propose ABs		
Feels able to encourage students to practice ABs		
Feels/would feel safe to implement Abs		
CLASS CHARACTERISTICS *	10.31 ± 1.75	11.71 ± 2.25
Think students behave/could behave well during Abs		
During the school day, there is time to do Abs		
It is possible/would be likely to implement ABs with its students		
SUPPORTING THE SCHOOL EDUCATION NETWORK *	14.55 ± 2.17	15.83 ± 2.93
The school is committed to promoting the health and well-being of students		
Parents of pupils support the implementation of ABs		
Colleagues support the implementation of ABs		
The school principal supports the implementation of the ABs		

Mean and standard deviation M ± SD of the sum of Likert scale for each domain: individual perceptions, class characteristics, and supporting the school education network (adapted version of Attitudes Towards Physical Activity, ATPA [42]). * *p* < 0.05.

**Table 3:** Teacher barriers.

	Non-Implementers (N = 49)	Implementers (N = 21)
BARR1 Space available is adequate	2.75 ± 1.02	2.38 ± 0.90
BARR2 No fear of injuries	3.32 ± 0.98	3.61 ± 0.90
BARR3 Material is sufficient	3.12 ± 0.77	3.38 ± 1.05
BARR4 Bureaucratic process for ABs is simple	3.10 ± 0.71	3.33 ± 0.99
BARR5 Class is too large	2.83 ± 0.96	3.09 ± 1.02
BARR6 Class is easy to manage	2.81 ± 0.96	2.61 ± 1.13
BARR7 Training is sufficient	3.16 ± 0.74	3.23 ± 1.02
BARR8 Time available is sufficient	3.14 ± 0.83	3.19 ± 0.85

Mean and standard deviation M ± SD values for items assessing potential barriers to ABs implementation, measured on a 5-point Likert scale. Comparisons are reported between Implementers and Non-Implementers. No significant differences were observed between groups (all *p* > 0.05).

**Table 4:** Teachers’ perceptions of the benefits of ABs.

	PRIMARY M ± SD	SECONDARY M ± SD
ABs help children sit more easily	4.02 ± 0.64	3.39 ± 0.82
ABs facilitate the achievement of learning objectives	3.29 ± 0.69	3.35 ± 0.68
ABs facilitate conflict management	3.54 ± 0.78	3.09 ± 0.70
ABs are simple and fun	4.32 ± 0.68	4.03 ± 0.85
ABs are more effective than other strategies	3.82 ± 0.60	3.56 ± 0.85
ABs improve the classroom environment	4.00 ± 0.71	3.71 ± 0.89
ABs facilitate behavior management	3.89 ± 0.84	3.47 ± 0.88

Mean and standard deviation M ± SD values for items assessing potential perceptions of benefits of AB implementation among teachers, measured on a 5-point Likert scale.

## Data Availability

The data presented in this study are not publicly available due to privacy or ethical restrictions. Data may be available from the corresponding author upon reasonable request and subject to approval by the relevant ethics committee.

## Abbreviations

The following abbreviations are used in this manuscript:

ABs	Active Breaks
ASD	Autism Spectrum Disorder
ATPA	Attitudes Towards Physical Activity
EFA	Exploratory Factor Analysis
GDPR	General Data Protection Regulation
IPAQ	International Physical Activity Questionnaire
KMO	Kaiser–Meyer–Olkin
METs	Metabolic Equivalents
PA	Physical Activity
SBs	Sedentary Behavior
SURGE	Survey Reporting Guideline

## Appendix A

**Table A1:** Teachers’ perceptions of the practicality, benefits, applicability and sustainability of ABs.

	Primary (N = 57)		Secondary (N = 34)	
**Did you feel comfortable suggesting active breaks?**	**N.**	**%**	**N.**	**%**
Definitely YES	44	77.19	24	70.59
More YES than NO	12	21.05	8	23.53
More NO than YES	1	1.75	2	5.88
**In which month did you start performing active breaks?**				
Autumn (September–November)	19	33.33	4	11.76
Winter (December–January–February)	23	40.35	23	67.65
Spring (March–April–May)	15	26.32	7	20.59
**How often did you perform active breaks?**				
A few times a week	29	50.88	22	64.71
A few times a month	5	8.77	7	20.59
Every day once a day	12	21.05	5	14.71
Every day several times a day	11	19.30	0	0.00
**How long does an active break usually last?**				
15 min	2	3.51	1	2.94
10 min	34	59.65	14	41.18
5 min	20	35.09	15	44.12
Others	1	1.75	4	11.76
**At what time of the school day is the active break most useful?**				
Early morning	16	28.07	4	11.76
Midmorning	25	43.86	26	76.47
After lunch break	6	10.53	0	0.00
Afternoon	8	14.04	0	0.00
Other	2	3.51	4	11.76
**What type of content did you use most frequently in active breaks?**				
Cognitive	0	0.00	2	5.88
Disciplinary	3	5.26	1	2.94
Motor	39	68.42	23	67.65
Relational	1	1.75	2	5.88
Mixed Content	14	24.56	6	17.65
**Who led the active breaks?**				
Teacher	38	66.67	14	41.18
Expert on educational project	1	1.75	0	0.00
Pupils	0	0.00	8	23.53
Special needs teacher	5	8.77	1	2.94
Teacher and Pupils	6	10.53	9	26.47
Mixed Figures	7	12.28	2	5.88
**During the active break did you use the interactive whiteboard?**				
No	49	85.96	28	82.35
Yes	8	14.04	6	17.65
**What digital teaching tools/applications have you used with the interactive whiteboard? ***				
YouTube	4	7.02	6	17.65
Mixed tools	4	7.02	2	5.88
None	49	85.96	28	82.36

Number of observations n. and percentage % across school levels. * multiple choice question.

**Table A2:** Inclusion and disability.

	Primary (N = 57)		Secondary (N = 34)	
**Were there pupils with disabilities in your class this year?**	**N.**	**%**	**N.**	**%**
No	20	35.09	10	29.41
Yes	34	59.65	22	64.71
I prefer not to answer	3	5.26	2	5.88
**Teachers who taught pupils with disabilities**	**(N = 34)**		**(N = 22)**	
**Can you specify the type of disability referred to the pupils in your class? ***				
Cognitive	27	79.41	15	68.18
Physical	0	0	2	9.09
Sensory	2	5.88	0	0
Cognitive and Physical	1	2.94	1	4.55
Cognitive and Sensory	2	5.88	0	0
I prefer not to answer	2	5.88	4	18.18
**Was it necessary to adjust the timing/rhythms/scheduling of the ABs to meet the needs of the students with disabilities? ***				
Never	11	32.25	10	45.45
Almost never	8	23.53	2	9.09
Rarely	1	2.94	2	9.09
Sometimes	10	29.41	6	27.27
Always	4	11.76	2	9.09

Number of observations n. and percentage %. Questions * were provided by a subset of participants only.
